# Supplementary material for: The Use of Different Anthropometric Indices to Assess the Body Composition of Young Women in Relation to the Incidence of Obesity, Sarcopenia and the Premature Mortality Risk
Source: Int J Environ Res Public Health. 2022 Sep 29;19(19):12449. doi: 10.3390/ijerph191912449 (PMC9564835; doi:10.3390/ijerph191912449)
Supplement: Supplementary file 1 [file ijerph-19-12449-s001.zip › ijerph-1878292-supplementary.pdf]

Supplementary Materials

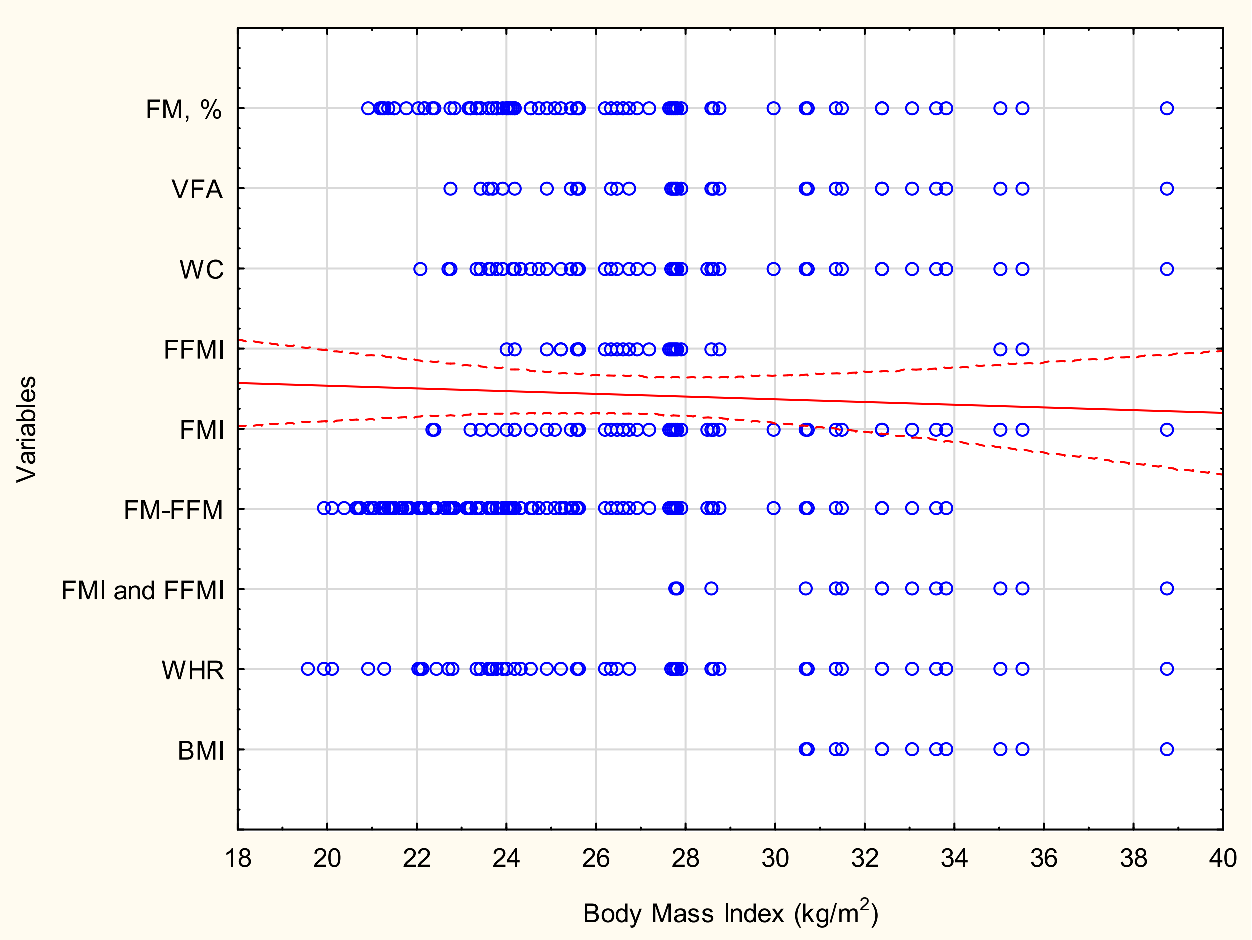

Figure S1. Variable-adjusted distribution of BMI values in young obese women.

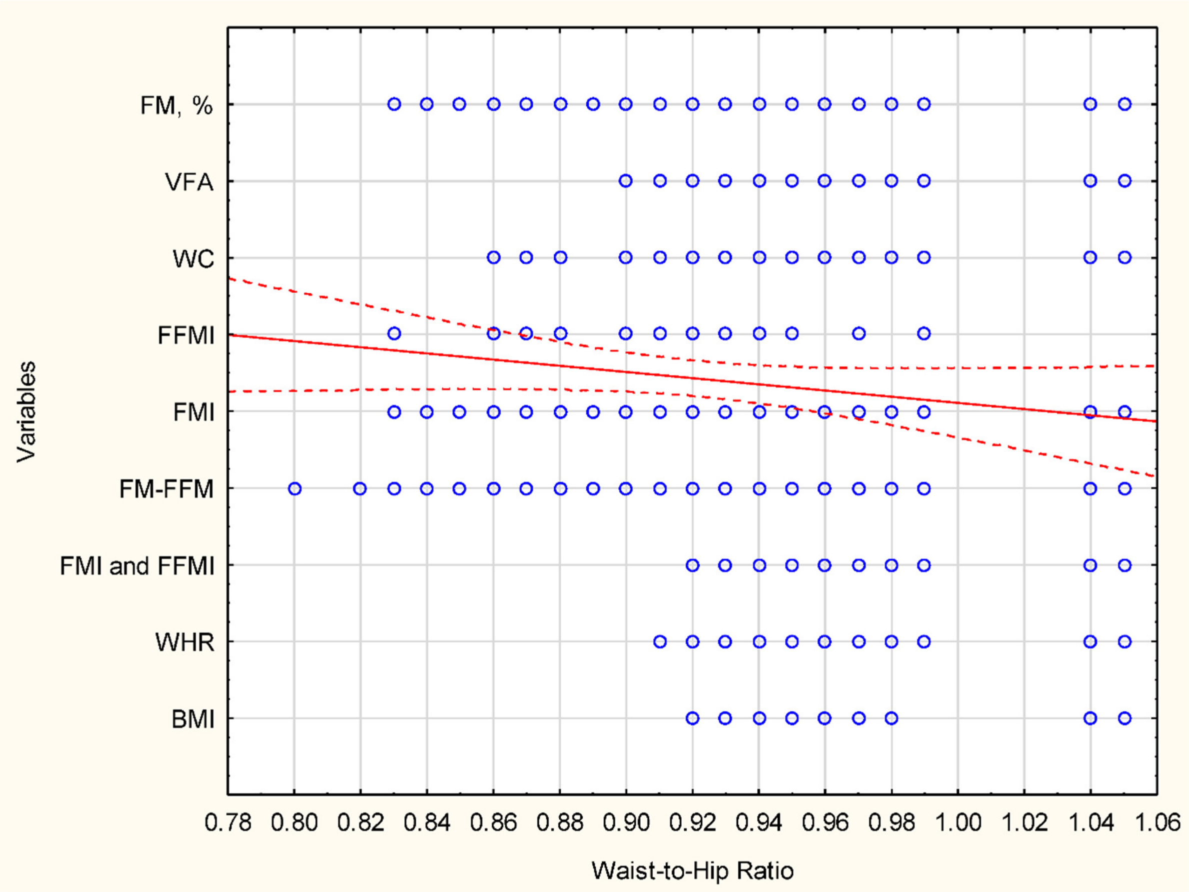

Figure S2. Variable-adjusted distribution of WHR values in young obese women.

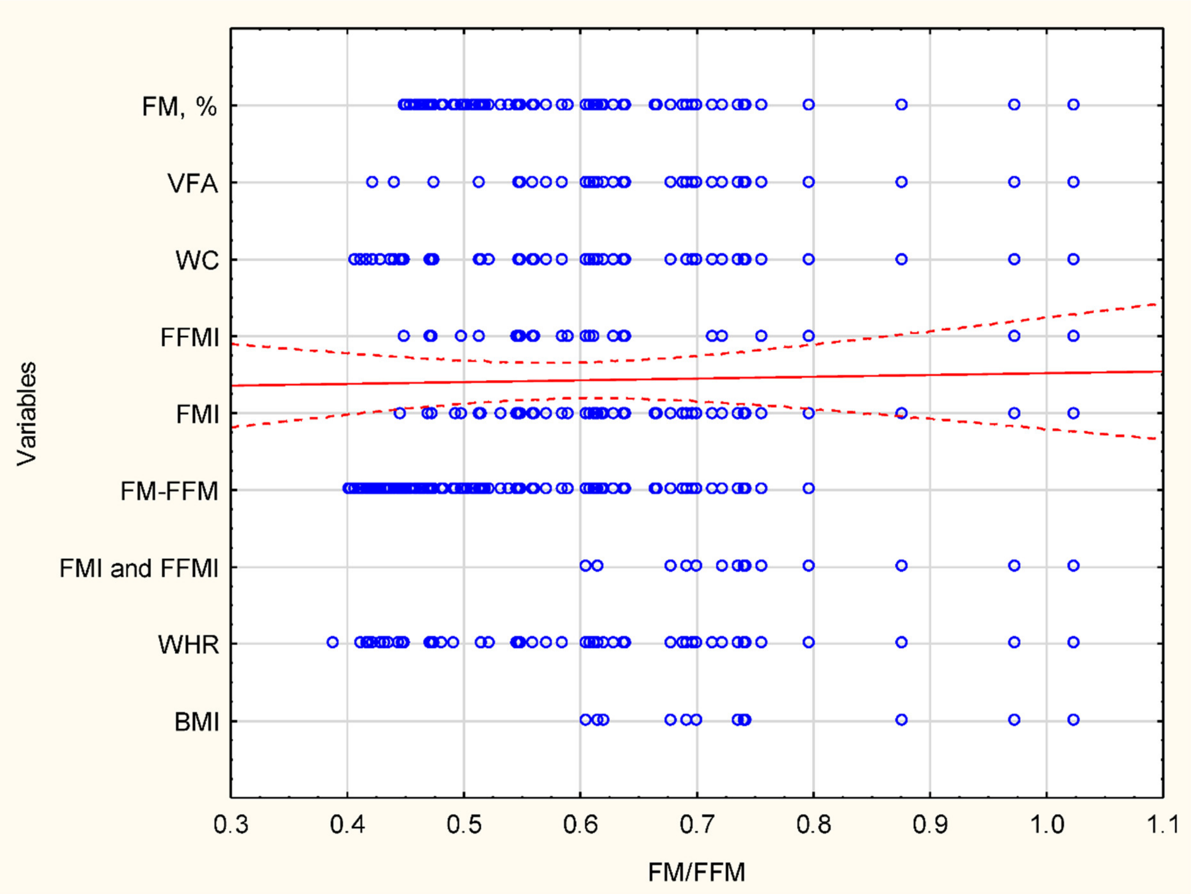

Figure S3. Variable-adjusted distribution of FM/FFM values in young obese women.

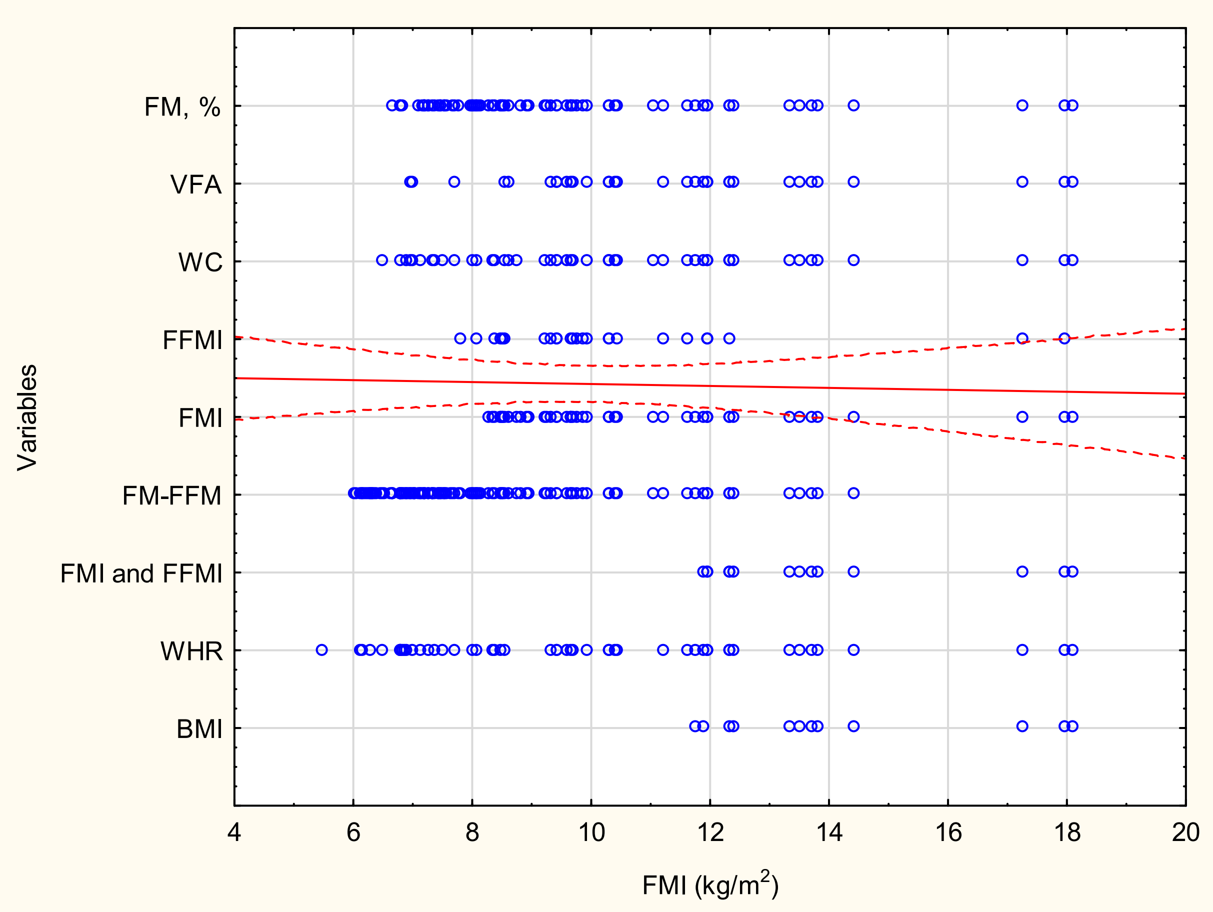

Figure S4. Variable-adjusted distribution of FMI values in young obese women.

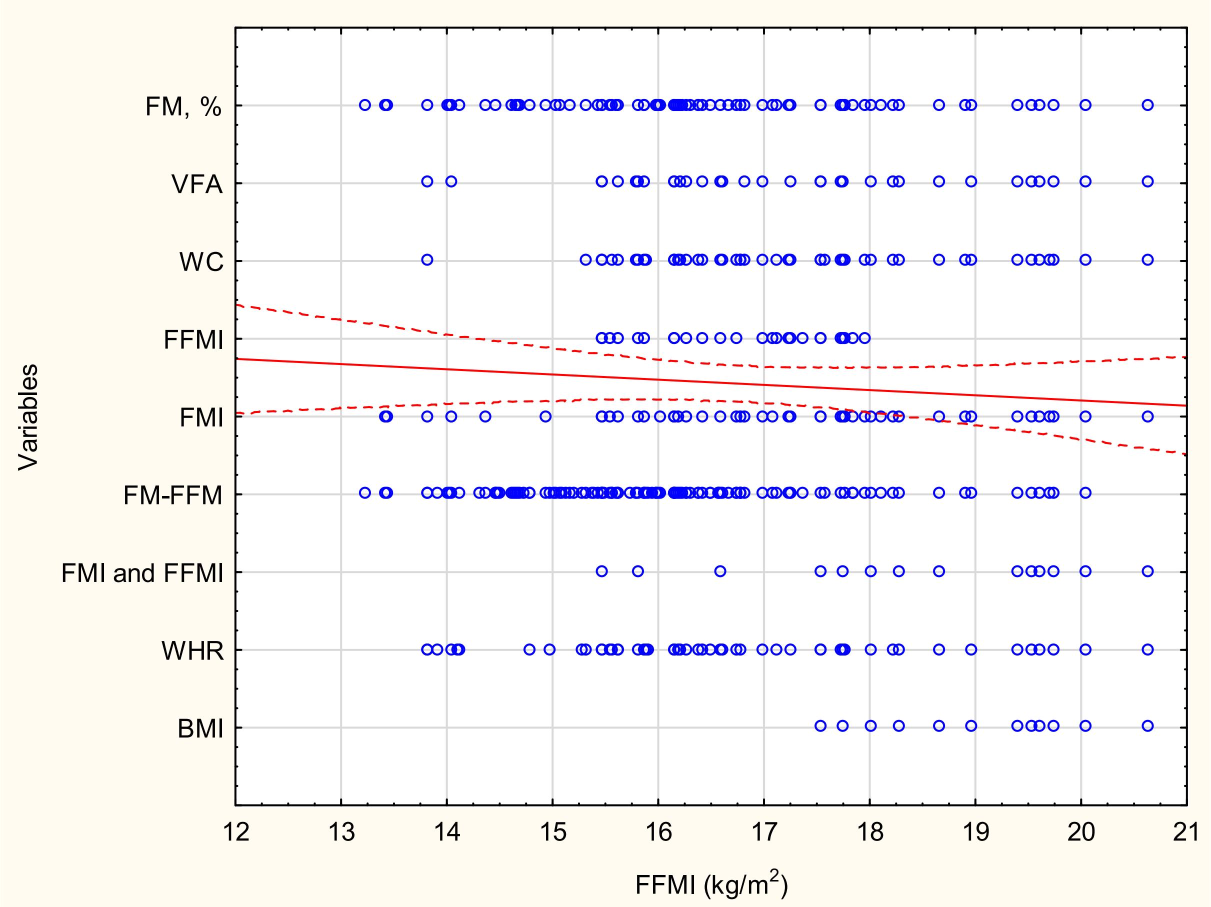

Figure S5. Variable-adjusted distribution of FFMI values in young obese women.

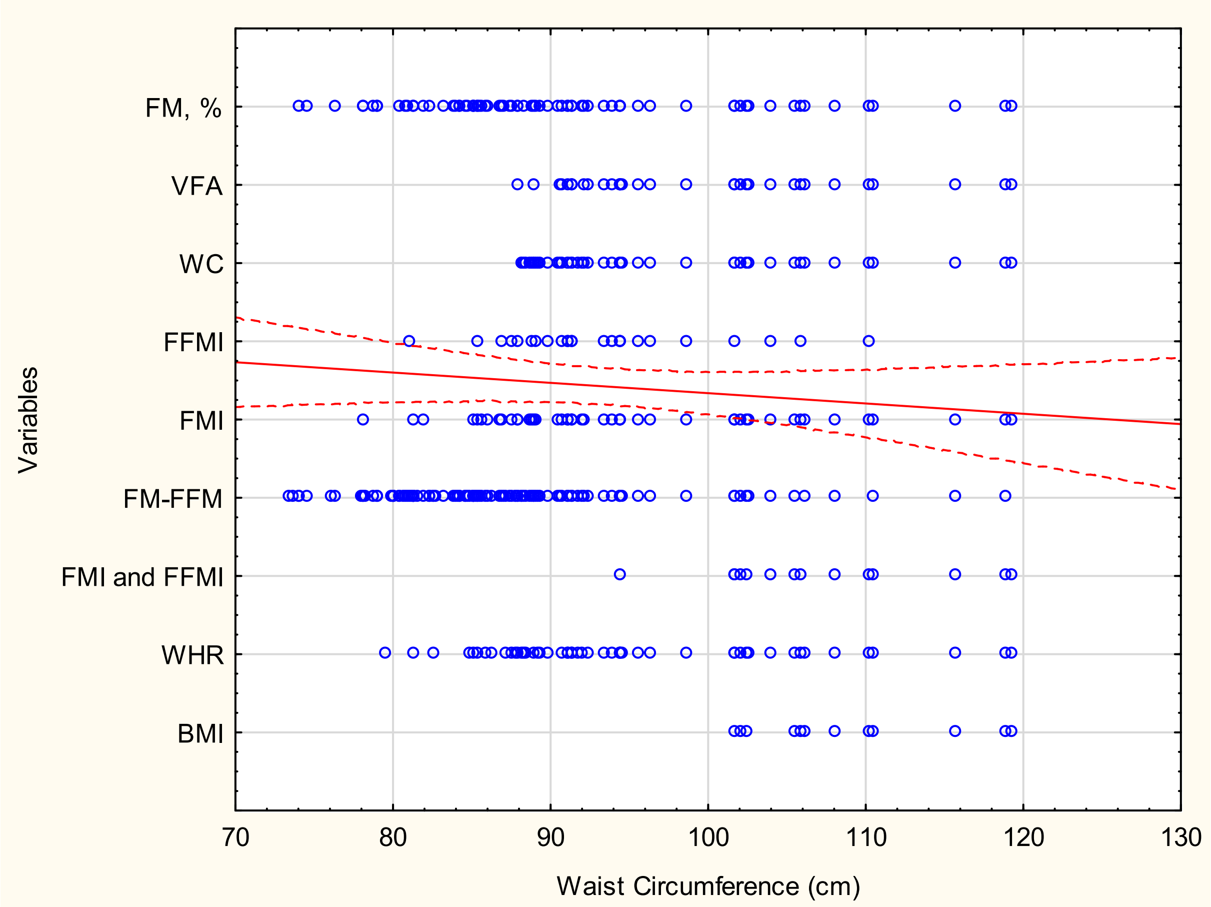

Figure S6. Variable-adjusted distribution of WC values in young obese women.

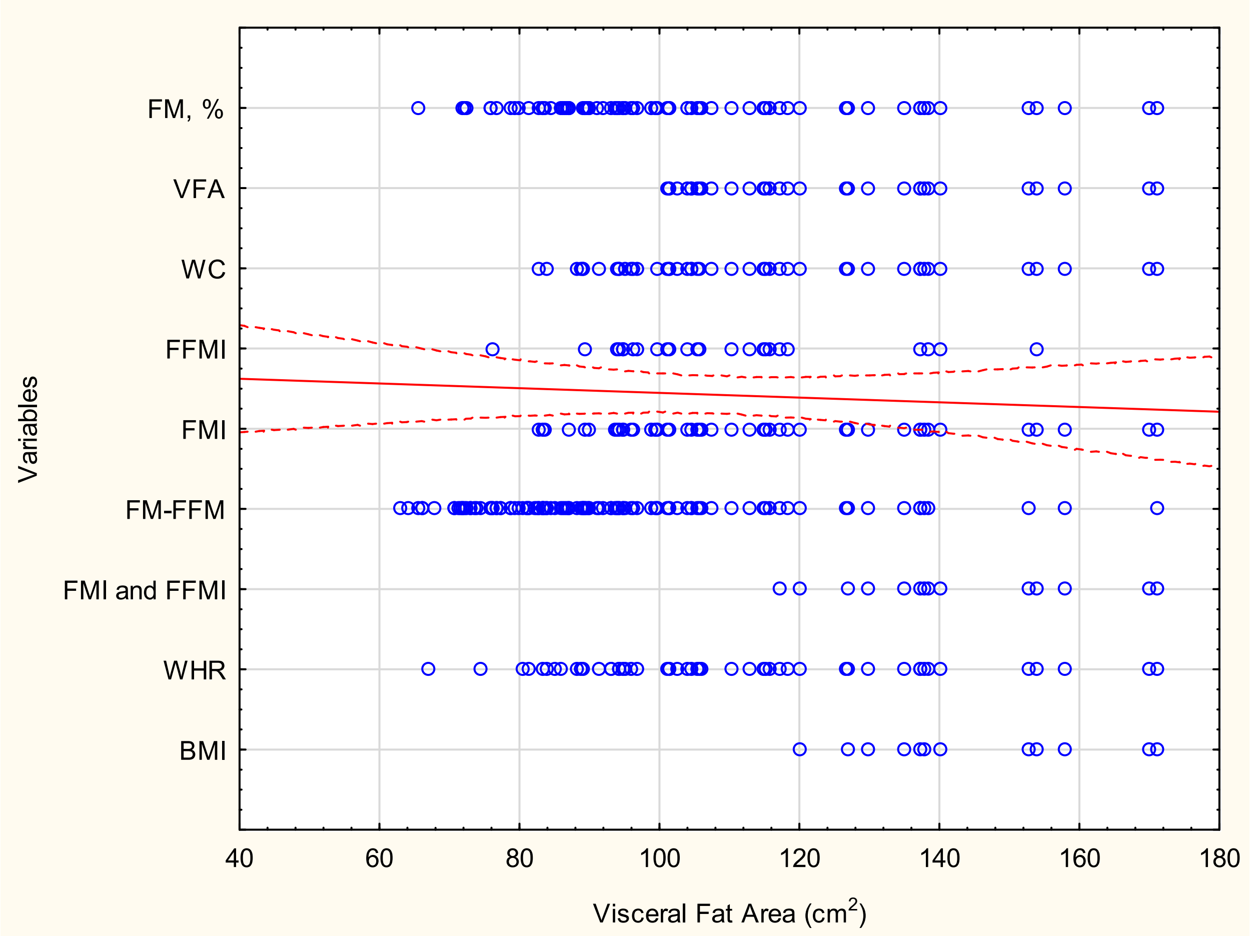

Figure S7. Variable-adjusted distribution of VFA values in young obese women.
